# The relationship between perceived AI replacement threat and employment anxiety among English-major undergraduates: the mediating role of career insecurity and the moderating role of career decision-making self-efficacy

**DOI:** 10.3389/fpsyg.2026.1867329

**Published:** 2026-10-27

**Authors:** Suojun Zhang, Hairong Zheng

**Affiliations:** 1 School of Foreign Languages, Quzhou University, Quzhou, Zhejiang, China; 2 School of Foreign Languages, Hulunbuir University, Hulunbuir, China

**Keywords:** career decision-making self-efficacy, career insecurity, employment anxiety, English-major undergraduates, perceived AI replacement threat

## Abstract

**Background:**

Concerns over possible employment displacement among undergraduates majoring in English have been raised by the growing use of artificial intelligence in writing, translation, and language services. However, limited research has examined the association between perceived AI replacement threat (PAIRT) and employment anxiety among English-major undergraduates or the roles of career insecurity and career decision-making self-efficacy (CDMSE).

**Methods:**

This cross-sectional study surveyed 711 Chinese English-major undergraduates recruited through convenience sampling. After controlling for gender and grade level, AMOS and PROCESS Model 7 with 5,000 bootstrap resamples were used to examine the indirect and conditional indirect associations.

**Results:**

PAIRT was positively associated with both career insecurity and employment anxiety. A significant indirect association between PAIRT and employment anxiety through career insecurity was observed, accounting for 48.29% of the estimated total effect. CDMSE significantly moderated the association between PAIRT and career insecurity, with this positive association becoming weaker at higher levels of CDMSE. The conditional indirect association through career insecurity was smaller at higher levels of CDMSE, and the index of moderated mediation was significant. Sensitivity analyses excluding the career replaceability items from employment anxiety produced substantively consistent results.

**Discussion:**

These findings extend understanding of employment anxiety among English-major undergraduates in the context of AI.

## Introduction

Against the dual backdrop of the continued growth of college graduates and the swift advancement of artificial intelligence, college students’ employment anxiety has become a real issue worthy of attention. According to official statistics, the number of graduates from China’s conventional universities in the class of 2025 was 12.22 million, up 430,000 from the year before, and employment pressure is still present (China T. S. C. o. t. P. s. R. o, 2025). Meanwhile, artificial intelligence is constantly changing the composition of job tasks and the skill requirements. According to a World Economic Forum report, 40% of firms anticipate a decrease in labor demand for jobs that AI can automate, while almost two-thirds of businesses want to hire workers with specialized AI capabilities (Forum W. E., 2025). Against this backdrop, the tension and anxiety encountered by college students while looking for a job and choosing a career, arising from uncertainties regarding job availability, skill-job fit, and career prospects, has become a psychological phenomenon that cannot be ignored (Liu and Zhang, 2025). For English-major undergraduates, the career fields they may enter in the future, such as translation, writing and language services, have a high degree of overlap with artificial intelligence application scenarios. Therefore, AI-related occupational change is particularly relevant to the career context of English-major undergraduates (Yaşar and Karagucuk, 2025). Based on this, given the speed at which artificial intelligence is developing, employment anxiety among English-major undergraduates therefore warrants attention in the context of AI-related occupational change.

Studies on college students’ anxiety related to their jobs have progressively broadened in recent years from a general focus on labor market uncertainty, competition, and employment pressures to a thorough analysis of new occupational environmental elements and individual psychological traits (Kwak et al., 2025; Li et al., 2025; Usanmaz et al., 2026). Research on the career psychology of college students indicates that perceptions of employment risks in the context of technological change are increasingly becoming a crucial element in understanding their employment anxiety. For example, there is a significant correlation between the impact of artificial intelligence as perceived by college students and the hazards associated with employment (Liang and Zhai, 2025). Recent research has examined AI-related anxiety in the career development of English-major undergraduates. This indicates that students majoring in English-related fields have begun to recognize the connection between the development of artificial intelligence and their own career prospects (Wang, 2026). In this context, concerns about replacement and perceived career threats related to the development of artificial intelligence have become important contextual factors in understanding employment anxiety among college students (Ge et al., 2025). At the same time, as college students move from campus to the labor market, they often worry about the stability and sustainability of their future careers. Therefore, career insecurity (CI) has gradually become a focus in related research (Kwak et al., 2025). In addition, career decision-making self-efficacy (CDMSE) is closely related to indicators of career adaptation, such as career anxiety, and studies have begun to focus on its moderating role in uncertain situations (Söner and Yılmaz, 2025).

However, most existing studies have examined college students’ career-related psychological adaptation from separate perspectives, such as AI-related anxiety, perceived employment risk, or career decision-making beliefs. In the backdrop of artificial intelligence taking the place of human labor, there is still a dearth of thorough study on how employment anxiety develops among English majors. In particular, the role of CDMSE within the connection between perceived AI replacement threat (PAIRT) and CI is still understudied in the body of current literature. Based on this, this study draws on cognitive appraisal theory (CAT) and focuses on undergraduate students majoring in English, with the aim of providing more context-specific evidence for research about college students’ concern about their jobs in the context of artificial intelligence.

### Theoretical framework

This study is based on the framework of CAT. The theory proposes that when faced with external environmental stress, individuals understand the meaning of events through cognitive evaluation processes and, based on this, form corresponding emotional responses (Lazarus and Folkman, 1984). Among them, primary appraisal mainly involves an individual’s evaluation of whether an external event is personally relevant and whether it represents harm/loss, threat, or challenge. Secondary appraisal entails a person’s additional evaluation of coping options, perceived control, and accessible coping resources. In recent years, CAT has been widely used in research on stress perception, risk judgment, and anxiety experience, and has gradually extended to the psychological context of college students’ career development and employment (Duan et al., 2026; Ge et al., 2025; Liang and Zhai, 2025; Turner et al., 2024). In this study, PAIRT can be seen as a person’s appraisal of the possibility that artificial intelligence may replace their future job or core work tasks; this aligns with the threat appraisal component of primary appraisal in CAT. CI concerns uncertainty about personal career continuity, opportunities, responsibilities, and job quality, reflecting what may be at stake in one’s future career. Together, these constructs address related but distinct aspects of primary appraisal: AI-specific replacement risk and the security of personal career prospects. Employment anxiety, in turn, reflects the negative emotional experience associated with employment-related concerns, such as worry, tension, and unease. In addition, CDMSE reflects an individual’s subjective judgment of their own career decision-making and coping abilities, and can be regarded as an important cognitive factor related to the assessment of coping resources, perceived control, and coping possibilities in the secondary appraisal process. Accordingly, CAT can provide an overarching theoretical framework for explaining the relationships among PAIRT, CI, CDMSE, and employment anxiety in this study.

### Literature review and hypotheses

#### Perceived AI replacement threat and employment anxiety

PAIRT refers to a person’s subjective appraisal of the possibility that AI may replace their future job or core work tasks (Wang and Lu, 2025). Recent studies have also examined individuals’ perceptions of AI-related job replacement risks from different perspectives. Gui et al. (2025) identified replacement-related AI awareness as one form of AI-depleted awareness, while Zhang et al. (2026) investigated university students’ perceptions of the threat of AI job replacement in connection to future career adjustment. Employment anxiety reflects the worry, tension, and unease experienced in anticipation of future employment difficulties involving personal ability, knowledge application, occupational prospects, and workplace relationships. An appraisal that AI may replace one’s future job may therefore be associated with this broader employment-related emotional experience. CAT states that people are more prone to have negative emotional responses, such as worry and anxiety when they interpret changes in their external environment as possible threats to their objectives, resources, or future chances (Lazarus and Folkman, 1984; Yeo and Ong, 2024). As generative AI keeps becoming more and more important in language-intensive tasks such as translation and text generation, the connection between the career preparation landscape for English majors and the advancement of AI technology has become increasingly apparent. This also makes PAIRT relevant to real-world research (Gmyrek et al., 2023). Previous studies have shown that students majoring in English-related fields are more inclined to recognize the career uncertainties caused by artificial intelligence during their career preparation process. Especially in areas such as translation and interpretation, students have a more pronounced concern about their future career prospects and professional competitiveness (Yaşar and Karagucuk, 2025). Accordingly, PAIRT may be positively associated with employment anxiety among English-major undergraduates. Based on this, the following hypothesis is proposed:

*H1*: PAIRT is positively associated with employment anxiety among English-major undergraduates.

#### The mediating role of career insecurity

CI generally refers to an individual’s subjective concern that future career continuity, employment quality and career development prospects may be threatened (Antonio and Chiesa, 2024). It encompasses uncertainty regarding future career continuity, employment opportunities, job responsibilities, and job quality. The uncertainty created by technological development is increasingly likely to be seen by people as a bad indication of future employment prospects as artificial intelligence continues to dominate the workplace. Existing research shows that people report feeling more insecure about their careers when they are more aware of intelligent technology, artificial intelligence, robots, and algorithms (Lingmont and Alexiou, 2020). Among English-major undergraduates, concerns about AI applications in translation, writing, and language services may be associated with uncertainty about future career opportunities and continuity.

Based on this, CAT posits that when individuals perceive external changes as a danger to their objectives, resources, or future development, they are more likely to experience negative emotional reactions such as anxiety (Lazarus and Folkman, 1984). Previous studies have demonstrated a consistent relationship between insecurity and anxiety, which is consistent with this theoretical reasoning. A meta-analysis found that job insecurity is significantly associated with detrimental psychological effects including anxiety, depression, and emotional exhaustion (Llosa et al., 2018). Subsequent research has also found that CI is accompanied by higher levels of anxiety reactions (Aguiar-Quintana et al., 2021). In relation to university students, external environmental factors and internal cognitive biases during the job search process have also been associated with employment anxiety (Wu et al., 2024). Therefore, for English major college students, when they lack confidence in their future career entry and job stability, an increased degree of CI is more likely to be accompanied by an increased degree of employment anxiety. Based on these considerations, the following hypotheses are proposed:

*H2*: PAIRT is positively associated with CI among English-major undergraduates.

*H3*: CI is significantly and positively associated with employment anxiety among English-major undergraduates.

*H4*: PAIRT shows a significant indirect association with employment anxiety through CI among English-major undergraduates.

#### The moderating role of career decision-making self-efficacy

CDMSE is a person’s subjective evaluation of their level of confidence in carrying out tasks related to career decision-making, such as gathering occupational data, comparing plans, and carrying out decisions (Wang et al., 2022). According to CAT, people evaluate whether they have enough resources, a sense of control, and the capacity to cope when confronted with external stressors in addition to determining whether the event poses a threat. This secondary appraisal is relevant to subsequent emotional responses (Lazarus and Folkman, 1984; Liu and Li, 2026). For English major college students, as artificial intelligence continues to enter language-related fields such as translation, writing and text processing, the technological changes are more likely to be incorporated into the assessment of their career prospects. Related research also shows that college students’ perceptions of artificial intelligence are positively associated with their perceived employment risk (Liang and Zhai, 2025). Against this backdrop, CDMSE may be relevant to appraisals of future career prospects. It reflects confidence in handling career exploration, choice, and adjustment, making it conceptually relevant to judgments of future career stability and controllability. Compared with general emotional reactions, it is first related to how individuals understand their own coping ability in career exploration, career choice and career adjustment, and is therefore more likely to be reflected in their judgment of future career stability and controllability. According to earlier studies, CDMSE is linked to more favorable career transition preparation and adaptive states (Söner and Yılmaz, 2025). Accordingly, when English-major undergraduates report higher levels of PAIRT, those with higher CDMSE are more likely to maintain relatively stable career evaluations based on stronger confidence in their abilities. In contrast, those with lower CDMSE are more likely to experience greater uncertainty in response to perceived threats. Accordingly, the following hypothesis is proposed:

*H5*: CDMSE moderates the association between PAIRT and CI.

Previous research has reported differences in employment anxiety across gender and grade levels among Chinese university students (Yang et al., 2025). Gender is relevant to differences in employment-related experiences and expectations, whereas grade level reflects students’ stage of career preparation and proximity to the transition from university to employment. Gender and grade level were therefore incorporated as variables in the subsequent analyses.

The research model is depicted in Figure 1.

**Figure 1:**
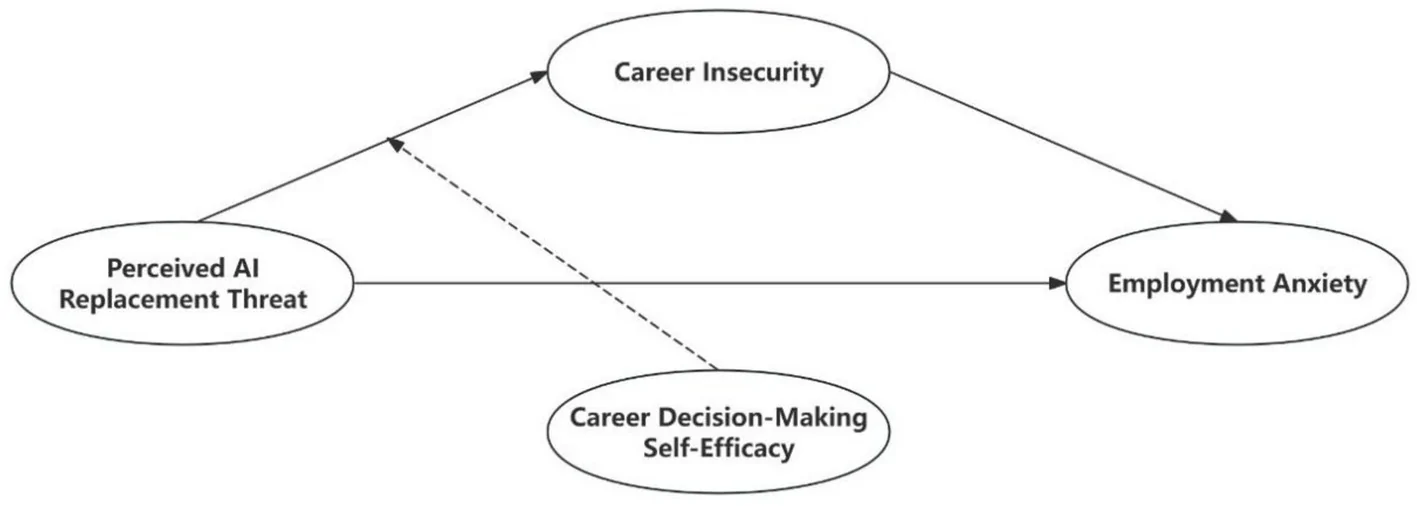
Research model.

## Methods

### Sample and data collection

This research was authorized by the ethics committee of Quzhou University (Approval No. QZXY-2026-12), addressing every facet of data gathering, data analysis, and subject recruiting. All procedures comply with human research guidelines. Convenience sampling was used in this study to select undergraduates majoring in English from seven universities in Inner Mongolia Autonomous Region, Zhejiang Province, Shandong Province, Henan Province, Hebei Province, and Chongqing Municipality, China. The participating universities were approached through existing academic contacts and based on the feasibility of conducting the survey. With the assistance of relevant departments or faculty members at the participating universities, the link to the questionnaire was shared across class groupings of English-major undergraduates. When designing the questionnaire, we also collected data on the respondents’ gender, age, grade level, and household registration location. All participants participated voluntarily and signed informed consent before filling out the questionnaire to ensure that the study maintained the participants’ privacy and autonomy while adhering to ethical norms. The survey was administered through Wenjuanxing during March and April of 2026^1^. The questionnaire contained 54 scale items and four demographic questions. A heuristic of 10 participants per scale item was used as a sample size reference (Kline, 2016), yielding a benchmark of 540 participants. The recruitment target was set at 696 to allow for invalid responses. There were 752 answers in all. After data screening, 711 valid responses were retained, corresponding to 94.5% of the submitted questionnaires. The criteria for deeming a questionnaire invalid include: (1) a completion rate of less than 80%; and (2) over 80% of the items with the greatest or lowest score. This is to avoid floor or ceiling effects caused by extreme response bias, which could otherwise compromise the accuracy of data analysis. Table 1 provides specifics about the participants’ basic demographics.

**Table 1:** Demographic distribution of sample data.

Variables	Category	Number	Percentage (%)
Gender	Male	120	16.88
	Female	591	83.12
Age	18–19	181	25.46
	20–22	220	30.94
	>22	310	43.60
Grade level	First-year	108	15.19
	Second-year	137	19.27
	Third-year	186	26.16
	Fourth-year	280	39.38
Household registration location	Urban	316	44.44
	Rural	395	55.56

### Measures

The questionnaire included demographic questions and measures of the focal constructs. All questionnaires were administered in Chinese. The Future Employment Anxiety Scale was originally developed in Chinese and was used in its original Chinese form. For PAIRT, CI, and CDMSE, the Chinese wording followed previously published Chinese applications of the corresponding measures. A number of questions were contextually modified to represent the future work circumstances of undergraduates majoring in English. Two bilingual researchers with backgrounds in psychology and foreign language education independently reviewed the adapted items, and a third bilingual researcher back-translated them into English. To preserve conceptual and semantic equivalency, differences between the back-translated and original English elements were discussed. All participants completed the final Chinese questionnaire.

This study employed the four-item scale developed by Brougham and Haar (2018) to measure PAIRT among English-major undergraduates. A sample item is “I think AI might replace the job I will do in the future.” The four items represent an overall perception that artificial intelligence may replace an individual’s future job or core work tasks. A five-point Likert scale was used to grade the items; higher scores corresponded to greater PAIRT levels. Previous research involving Chinese university students has supported the reliability and validity of this measure (Liang and Zhai, 2025). Cronbach’s alpha for this measure was 0.702.

This study used the Future Employment Anxiety Scale developed by Wang et al. (2025) to assess employment anxiety among English-major undergraduates. The scale contains 17 items and comprises four related dimensions: personal ability (PA, five items), knowledge application (KA, four items), career replaceability (CR, four items), and social relations (SR, four items). A sample item is “I’m concerned that my expertise isn’t as strong as other people’s.” Together, these four dimensions represent students’ overall anxiety concerning their future employment. Accordingly, the mean of all 17 items was used as the composite employment anxiety score in the descriptive, correlational, and PROCESS analyses. A five-point Likert scale was used to grade the items; higher scores denoted higher levels of work-related anxiety. The Cronbach’s alpha coefficients for PA, KA, CR, and SR were 0.905, 0.864, 0.856, and 0.895, respectively. Cronbach’s alpha for the overall scale was 0.944.

Following the measurement approach used by Jia et al. (2022), CI was assessed using eight items. Four items adapted from De Witte (2000) address quantitative CI, whereas four items adapted from Probst et al. (2020) address qualitative CI. These items capture two related aspects of students’ overall uncertainty regarding their future careers. Although quantitative and qualitative CI reflect different facets of future employment uncertainty, they jointly represent individuals’ overall perceptions of CI. Therefore, the composite score of the eight items was used to assess overall CI in the present study. Quantitative CI concerns the continuity and stability of future employment, whereas qualitative CI concerns uncertainty regarding future career opportunities, job responsibilities, and job quality. The wording was contextually adapted to reflect the future employment situation of English-major undergraduates. A sample item is “I think it might be difficult for me to find a job related to my English major after graduation.” In the descriptive, correlational, and PROCESS analyses, total CI was represented by the mean of the eight items. A five-point Likert scale was used to grade each item; higher scores denoted greater degrees of career uncertainty. The Cronbach’s alpha coefficients for the quantitative and qualitative components were 0.823 and 0.806, respectively, and Cronbach’s alpha for the overall scale was 0.860.

This study used the 25-item CDMSE-SF developed by Betz et al. (1996). A sample item is “I can use the internet to search for information about English-related careers that interest me, such as translation, education, and international business.” The items cover five career decision-making task domains: self-appraisal, occupational information, goal selection, planning, and problem solving, with five items addressing each domain. These task domains reflect related aspects of students’ confidence in completing career decision-making activities. Although these domains represent theoretically distinct career decision-making tasks, previous validation research among Chinese students indicated that the five dimensions were highly homogeneous. Specifically, Jin et al. (2012) found that a parsimonious single-factor model showed comparable fit to multidimensional models, and the subdimensions did not provide substantial incremental validity beyond the overall score. Therefore, a total score representation was recommended when assessing overall career decision-making self-efficacy among Chinese student samples. Accordingly, the overall mean score was used to represent CDMSE in the present study. A five-point Likert scale was used to grade the items; higher scores indicated more confidence in finishing tasks related to career decision-making. The validity and reliability of the measure among Chinese university students have been confirmed by earlier studies (Zhang et al., 2024). The Cronbach’s alpha coefficients for self-appraisal, occupational information, goal selection, planning, and problem solving were 0.826, 0.819, 0.798, 0.822, and 0.835, respectively. Cronbach’s alpha for the overall scale was 0.959.

### Data analysis

Data analyses were conducted using SPSS 26.0, AMOS 29.0, and PROCESS 4.2. SPSS was used to calculate descriptive statistics, Pearson correlations, internal consistency coefficients, and univariate normality indices. Potential common method bias was examined using Harman’s single-factor test and a common latent factor (CLF) approach. Confirmatory factor analysis (CFA) was conducted in AMOS 29.0 before the hypothesized associations were examined. Measurement specifications were evaluated according to the theoretical structures of the instruments and available psychometric evidence. Because correlated-factor and higher-order models represent different forms of multidimensionality, competing measurement models were compared where applicable (Dunn and McCray, 2020). PAIRT was specified as a single first-order construct. For employment anxiety, one-factor, correlated four-factor, and second-order models were compared. The second-order structure of employment anxiety was maintained in all CFA model comparisons. For CI, one-factor and correlated two-factor models were compared. CDMSE was represented as a single overall construct based on previous evidence from Chinese student samples (Jin et al., 2012). The final measurement specification was subsequently used in the structural model. Gender and grade level were included as covariates based on their substantive relevance to employment anxiety and the focal variables (Bernerth and Aguinis, 2016; Spector and Brannick, 2011; Yang et al., 2025). For the PROCESS analyses, composite mean scores were calculated according to the scoring procedures described in the Measures section. Continuous focal variables were standardized before model estimation. Gender and grade level were included as covariates, and CDMSE was specified as the moderator in the first-stage moderation model. The conditional indirect association was investigated using PROCESS Model 7 with 5,000 bootstrap resamples.

## Results

### Descriptive statistics and correlations

Table 2 displays Pearson correlations and descriptive statistics. Absolute skewness and kurtosis values were below 2 and 7, respectively, suggesting no severe departures from univariate normality (Kline et al., 2011). PAIRT was significantly and positively correlated with employment anxiety and CI. CDMSE was significantly and negatively correlated with the other focal variables.

**Table 2:** Descriptive statistics and correlations among the study variables.

Variable	M ± SD	SK	Kur	PAIRT	EA	CI	CDMSE
PAIRT	3.208 ± 0.758	−0.142	0.656	1			
EA	3.413 ± 0.711	−0.333	0.966	0.551***	1		
CI	3.216 ± 0.785	−0.124	0.118	0.450***	0.677***	1	
CDMSE	2.454 ± 0.593	1.123	4.152	−0.112**	−0.177***	−0.187***	1

**p < 0.01; ***p < 0.001; PAIRT, perceived AI replacement threat; EA, employment anxiety; CI, career insecurity; CDMSE, career decision-making self-efficacy.

### Common method bias

Potential common method bias was examined using Harman’s single-factor test and a CLF approach. Firstly, the results of exploratory factor analysis revealed that the variance explained by the first factor was 27.429%, below the 40% criterion for an empirical critical value, and that there were five factors with eigenvalues greater than 1 (Podsakoff and Organ, 1986), which suggests that the problem of common methodological bias may not be serious. Second, in CFA, all observed variables are loaded simultaneously onto their respective theoretical constructs and a CLF. The findings demonstrated that the chi-square difference did not reach a significant level in the baseline model compared to the model after the inclusion of the CLF. This indicates that adding the CLF did not significantly improve the model fit. These analyses did not indicate substantial common method bias, although shared method variance cannot be ruled out.

### Reliability and validity of the model

Before examining the structural relationships, the measurement model was evaluated by assessing reliability and convergent validity. As presented in Table 3, most standardized factor loadings exceeded the recommended threshold of 0.70, although several items showed slightly lower loadings. These items were retained because they did not substantially affect the overall reliability and validity of the corresponding constructs. The Cronbach’s alpha coefficients ranged from 0.702 to 0.959, indicating acceptable internal consistency. In addition, the composite reliability values ranged from 0.816 to 0.962, exceeding the recommended criterion of 0.70. All average variance extracted (AVE) values were above 0.50, supporting the convergent validity of the measurement model (Henseler et al., 2015).

**Table 3:** Reliability and convergent validity of the measurement model.

Construct	Dimension	Items	Item loading	Second-order loading	α	Composite reliability	AVE
PAIRT	—	PAIRT1-4	0.649–0.827	—	0.702	0.816	0.528
EA	PA	EA1-5	0.763–0.880	0.780	0.905	0.907	0.662
	KA	EA6-9	0.717–0.854	0.896	0.864	0.872	0.630
	CR	EA10-13	0.681–0.835	0.875	0.856	0.859	0.604
	SR	EA14-17	0.769–0.864	0.803	0.895	0.897	0.686
Overall EA	Four first-order dimensions	—	—	—	0.944	0.905	0.705
CI	—	CI1-8	0.674–0.729	—	0.860	0.890	0.504
CDMSE	—	CDMSE1-25	0.588–0.783	—	0.959	0.962	0.506

PAIRT, perceived AI replacement threat; EA, employment anxiety; CI, career insecurity; CDMSE, career decision-making self-efficacy; PA, personal ability; KA, knowledge application; CR, career replaceability; SR, social relations.

### Confirmatory factor analysis

The measurement structure of the four focus constructs was assessed using CFA. PAIRT and CDMSE were represented as first-order constructs. Employment anxiety was represented as a second-order construct comprising four first-order dimensions: PA, KA, CR, and SR. CI was represented as an overall first-order construct. As seen in Table 4, the resulting measurement model demonstrated a satisfactory fit to the data.

**Table 4:** Model fit indices.

Fit index	Reference value	Final model
CMIN/DF	< 5.00	2.630
CFI	> 0.90	0.910
TLI	> 0.90	0.904
IFI	> 0.90	0.911
RMSEA	< 0.08	0.048
AIC	—	3923.482

For employment anxiety, the correlated four-factor model of employment anxiety and the second-order model both showed good fit. Although the correlated four-factor model of employment anxiety had a slightly lower χ^2^ and AIC, the differences in incremental fit indices were minimal (ΔCFI = 0.002; ΔTLI = 0.002), and the two models yielded the same RMSEA. The second-order model was retained because it represented both the four first-order dimensions and the overarching employment anxiety construct. For CI, the correlated two-factor model showed a modest improvement in fit over the one-factor model. However, the one-factor model also demonstrated good absolute fit (χ^2^/df = 2.292, CFI = 0.975, TLI = 0.961, RMSEA = 0.043), and the two dimensions were strongly correlated (r = 0.74). Given the theoretical focus on overall CI and the use of an overall composite score in the subsequent analyses, the more parsimonious one-factor specification was retained (Appendix Table S1).

### Construct distinctiveness

Given the conceptual proximity among PAIRT, employment anxiety, and CI, alternative measurement models were compared to further examine construct distinctiveness. As shown in Appendix Table S2, the hypothesized four-construct measurement model provided a better fit to the data than models in which PAIRT, employment anxiety, and CI were combined in different ways. The one-factor model showed particularly poor fit. These findings provided further evidence that the focal constructs could be distinguished empirically.

### Collinearity test

VIFs were calculated separately for the predictors included in each regression equation. As shown in Table 5, VIF values ranged from 1.009 to 1.315, all below the threshold of 3.3. These results indicated no substantial multicollinearity among the predictors.

**Table 5:** Collinearity test.

Outcome variable	Predictor	VIF
EA	PAIRT	1.309
	CI	1.315
	Gender	1.016
	Grade	1.009
CI	PAIRT	1.018
	CDMSE	1.027
	PAIRT × CDMSE	1.012
	Gender	1.016
	Grade	1.010

PAIRT, perceived AI replacement threat; EA, employment anxiety; CI, career insecurity; CDMSE, career decision-making self-efficacy.

### Structural model

#### Path hypotheses

Table 6 presents the structural model estimates with gender and grade level included as control variables. The results showed that PAIRT was significantly and positively associated with CI and employment anxiety, and CI was also positively associated with employment anxiety. Thus, all hypothesized direct paths were supported. Among the control variables, gender was significantly associated with employment anxiety, whereas the grade paths were not significant.

**Table 6:** Path estimates of the structural model (*N* = 711).

Path	Estimate	S. E.	C. R.	95% CI	*p*	Results
PAIRT → CI	0.743	0.076	9.715	[0.597, 0.924]	< 0.001	Supported
CI → EA	0.512	0.048	10.573	[0.408, 0.637]	< 0.001	Supported
PAIRT → EA	0.406	0.058	7.013	[0.275, 0.566]	< 0.001	Supported
Gender → CI	0.092	0.066	1.404	[−0.037, 0.221]	0.160	—
Gender → EA	0.171	0.045	3.816	[0.080, 0.276]	< 0.001	—
Grade → CI	0.001	0.023	0.062	[−0.043, 0.046]	0.951	—
Grade → EA	−0.01	0.015	−0.649	[−0.040, 0.017]	0.517	—

PAIRT, perceived AI replacement threat; EA, employment anxiety; CI, career insecurity. Gender was coded as 1 = male and 2 = female; grade was coded as 1 = first-year, 2 = second-year, 3 = third-year, and 4 = fourth-year.

#### Mediation analysis

Using 5,000 bootstrap resamples and 95% confidence intervals, the statistical indirect effect through CI was investigated. As shown in Table 7, both the direct effect and the indirect effect of PAIRT on employment anxiety were significant after controlling for gender and grade level. The estimated indirect effect was 0.380, representing 48.29% of the estimated total effect. These findings are consistent with the proposed mediation model.

**Table 7:** Test of the indirect effect of CI in the association between PAIRT and employment anxiety (*N* = 711).

Path	Estimate	SE	Bootstrapping				*p*	Effect proportion
			Bias-corrected 95% CI		Percentile 95% CI			
Indirect effect	0.380	0.057	0.285	0.514	0.275	0.500	< 0.001	48.29%
Direct effect	0.406	0.074	0.275	0.566	0.279	0.570	< 0.001	51.71%
Total effect	0.787	0.091	0.627	0.979	0.626	0.977	< 0.001	100.00%

### Moderation and conditional indirect effect analysis

After controlling for gender and grade level, the model predicting CI explained 23.1% of the variance (R^2^ = 0.231). As shown in Table 8, the interaction between PAIRT and CDMSE was significantly associated with CI. The interaction term accounted for an additional 0.6% of the variance in CI (*ΔR^2^* = 0.006, *F* (1, 705) = 5.783, *p* = 0.016). These results indicate that the association between PAIRT and CI varied significantly across levels of CDMSE.

**Table 8:** Moderation analysis (*N* = 711).

Variable	Estimate	SE	*t*	*p*	95% CI
PAIRT	0.435	0.033	13.077	< 0.001	[0.370, 0.501]
CDMSE	−0.127	0.033	−3.780	< 0.001	[−0.192, −0.061]
PAIRT × CDMSE	−0.051	0.021	−2.405	0.016	[−0.092, −0.009]
Gender	0.158	0.089	1.779	0.076	[−0.016, 0.332]
Grade	0.001	0.031	0.017	0.986	[−0.059, 0.060]

PAIRT, perceived AI replacement threat; CDMSE, career decision-making self-efficacy. Continuous focal variables were z-standardized before model estimation. The reported coefficients are the regression coefficients obtained from PROCESS based on these standardized variables. Gender was coded as 1 = male and 2 = female; grade was coded as 1 = first-year, 2 = second-year, 3 = third-year, and 4 = fourth-year.

Simple-slope analyses were further conducted to examine the pattern of the interaction (Figure 2). As shown in Table 9, the corresponding simple slopes were 0.486, 0.435, and 0.385 at low, mean, and high levels of CDMSE, respectively. Thus, the positive association between PAIRT and CI was weaker at higher levels of CDMSE. The model predicting employment anxiety explained 54.8% of the variance (R^2^ = 0.548).

**Figure 2:**
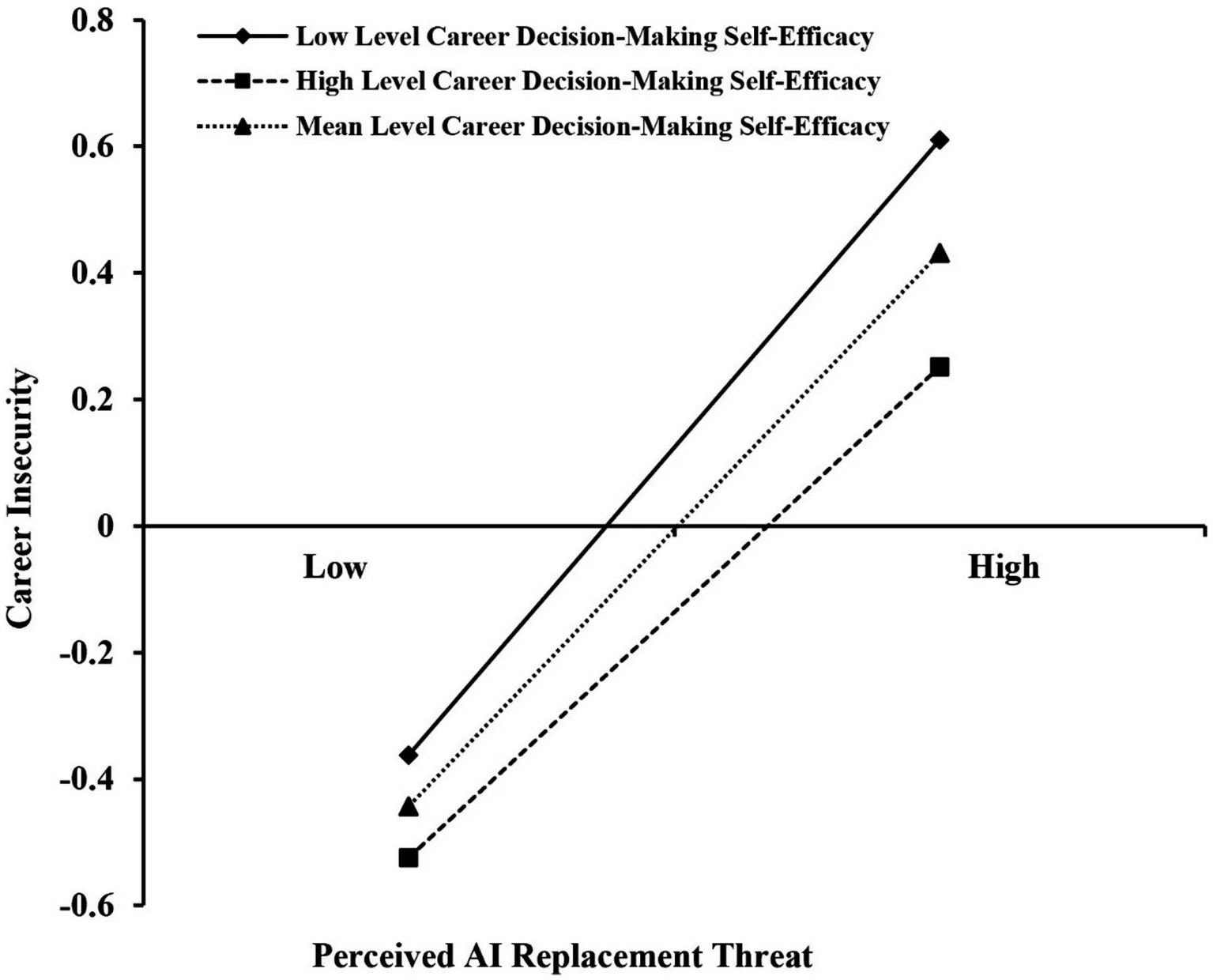
Moderating role of CDMSE. Low, mean, and high levels of CDMSE represent - 1 SD, the mean, and + 1 SD, respectively.

**Table 9:** Simple slopes, conditional indirect effects, and index of moderated mediation.

CDMSE level	Effect/Index	SE/BootSE	LLCI	ULCI	*p*
Simple slope: PAIRT → CI					
−1 SD	0.486	0.040	0.407	0.565	< 0.001
Mean	0.435	0.033	0.370	0.501	< 0.001
+1 SD	0.385	0.039	0.309	0.461	< 0.001
Conditional indirect effect: PAIRT → CI → EA					
−1 SD	0.257	0.029	0.199	0.315	—
Mean	0.231	0.026	0.180	0.281	—
+1 SD	0.204	0.029	0.144	0.259	—
Index of moderated mediation					
CDMSE	−0.027	0.014	−0.055	−0.002	-

PAIRT, perceived AI replacement threat; EA, employment anxiety; CI, career insecurity; CDMSE, career decision-making self-efficacy. Low, mean, and high levels of CDMSE correspond to −1 SD, the mean, and +1 SD, respectively.

The conditional indirect association between PAIRT and employment anxiety through CI was statistically significant at low, mean, and high levels of CDMSE (Table 9). The moderated mediation index was −0.027, and zero was not included in its 95% bootstrap confidence interval [−0.055, −0.002]. This result indicates that the statistical indirect association through CI varied significantly across levels of CDMSE.

### Robustness check

As an additional robustness check, the focal models were re-estimated with gender, age, grade level, and household registration included as covariates. As shown in Appendix Table S3, the estimates and 95% confidence intervals for the focal direct and indirect effects, interaction term, and index of moderated mediation were highly consistent with those obtained in the primary analyses. These results provide additional support for the robustness of the main findings to the inclusion of additional demographic covariates.

### Sensitivity analysis for potential construct overlap

A further sensitivity analysis was conducted to examine whether the inclusion of the CR dimension of employment anxiety influenced the observed associations involving PAIRT. In this analysis, the CR dimension was excluded, and the remaining 13-item employment anxiety scale was used to re-estimate the measurement model and structural associations. The CFA results of the revised employment anxiety scale showed acceptable model fit (χ^2^/df = 2.650, CFI = 0.914, TLI = 0.908, RMSEA = 0.048), indicating an acceptable measurement structure after excluding the CR dimension. The sample, analytical procedures, and adjustment for gender and grade level remained unchanged.

After excluding this dimension, the focal associations of PAIRT with CI and employment anxiety, as well as the statistical indirect effect through CI, remained significant. Their directions and magnitudes were broadly consistent with those in the main model. Further conditional process analyses showed that the interaction between PAIRT and CDMSE, simple slopes, conditional indirect effects, and index of moderated mediation retained the patterns observed in the main analyses. Overall, these findings provide additional support for the robustness of the main findings after excluding the CR dimension of employment anxiety. Detailed results are presented in Appendix Table S4–S7.

## Discussion

Drawing on CAT, this study examined a moderated mediation model among English-major undergraduates. PAIRT was positively associated with employment anxiety, and a significant statistical indirect effect through CI was observed. The positive association between PAIRT and CI was weaker at higher levels of CDMSE. The conditional indirect association through CI also varied across levels of CDMSE. These findings are consistent with the proposed model, although the cross-sectional design does not establish the temporal ordering of the associations.

PAIRT was significantly and positively associated with employment anxiety. In this study, on the one hand, the negative expectations of English majors that AI may replace some of their future occupational roles, that industry prospects may become more constrained, and that employment opportunities may decrease may be reflected in a stronger tendency to interpret technological changes as uncertain signals related to their own career prospects, and such threat-related appraisals may be associated with higher levels of employment anxiety (Lingmont and Alexiou, 2020; Savolainen et al., 2026). On the other hand, as English majors keep abreast of developments in artificial intelligence and its applications in fields such as translation and language services, they may feel pressure stemming from the potential for their professional roles to be replaced. In particular, greater awareness of the overlap between AI capabilities and language-related occupational tasks may be associated with stronger concerns about the future relevance of their professional skills and employment opportunities, which may correspond with higher levels of anxiety during job-search preparation, career choice, and future career adaptation. From the perspective of CAT, this pattern supports the idea that appraisals of potential threats to personally important career goals and resources are associated with negative emotional experiences such as tension and anxiety (Lazarus and Folkman, 1984). Related studies have also indicated that there is a consistent association between a sense of skill mismatch and workplace stress and anxiety levels (Christiansen et al., 2014; Jung et al., 2024), which provides further support for the findings of this study.

A significant statistical indirect association between PAIRT and employment anxiety through CI was observed. In this study, PAIRT may not be limited to a general appraisal of technological change but may also coexist with more concrete concerns about personal career prospects. English majors are more likely to reevaluate whether they will still be able to enter these industries, obtain stable positions, and maintain their career growth in the future when they perceive that artificial intelligence is making inroads into traditional English-related fields like translation, education, and language services (Parviz and Arthur, 2025; Yaşar and Karagucuk, 2025). From this perspective, an AI-specific perception of possible occupational replacement may be accompanied by broader concerns about career continuity, accessibility, and future opportunities, which are more directly represented by CI. Furthermore, compared to a general sense of environmental change, CI is more closely tied to an individual’s realistic assessment of post-graduation career paths, job fit, and long-term development. As such, higher CI may also be accompanied by greater employment-related anxiety, unease, and worry. Research has demonstrated that insecurity is frequently linked to people losing control and certainty over their future professional development, and that this pessimistic outlook is frequently accompanied by elevated levels of psychological stress and anxiety (Antonio et al., 2025; Kim and Kim, 2024).

CDMSE moderated the association between PAIRT and CI. The positive association was stronger at lower levels of CDMSE and weaker at higher levels of CDMSE, although it remained significant across the examined levels. When English-major undergraduates report lower CDMSE, they may have less confidence in obtaining occupational information, evaluating alternative career options, and planning how to respond to changing skill requirements. In this situation, concerns that AI may replace translation, writing, or other language-related work may be more readily associated with broader uncertainty about whether suitable jobs will remain available and whether their future careers can remain stable. PAIRT may therefore show a closer association with CI. In contrast, students with higher CDMSE may be more confident in identifying alternative occupations, comparing career paths, and adjusting their preparation to changing job requirements (Karna et al., 2024; Lee et al., 2022; Levin et al., 2024). AI-related changes may consequently be viewed not only as signals of possible job loss, but also as changes for which alternative directions and preparation strategies can be considered. This may explain the relatively weaker association between PAIRT and CI at higher levels of CDMSE. However, the moderating role of CDMSE should not be overstated. The interaction accounted for only an additional 0.6% of the variance in CI, and the association between PAIRT and CI remained significant even at higher levels of CDMSE. Thus, CDMSE appears to provide only a modest attenuation of this association and may not fully offset career uncertainty associated with PAIRT.

This moderating pattern is also meaningful for the indirect association through CI. For students who are less confident in making career decisions, concerns about AI replacement may be more likely to become embedded in a broader sense of uncertainty about their future careers. Once AI-related concerns are experienced in this broader form, they are no longer limited to whether a particular job or task may change, but become relevant to more immediate questions about entering the labor market, finding a suitable position, and maintaining a viable career direction. These worries are strongly related to the job anxiety that students have as they get ready to leave college and enter the workforce. In contrast, when students have greater confidence in making career decisions, AI-related replacement concerns may be less closely connected with this broader pattern of career uncertainty. The conditional indirect findings therefore suggest that CDMSE is relevant to the extent to which an AI-specific occupational concern is embedded within a wider pattern of CI and employment anxiety.

## Implications

### Theoretical implications

This study extends the application of CAT to anticipated career uncertainty among English-major undergraduates who have not yet entered the labor market. It highlights the theoretical relevance of distinguishing technology-specific threats from broader appraisals of personal career security when considering employment-related emotions. This distinction offers a more differentiated account of how threat appraisal can be conceptualized in AI-related career contexts. The consideration of CDMSE further adds a boundary-condition perspective, suggesting that the relevance of threat appraisal to CI may differ according to confidence in handling career decisions.

### Practical implications

For English-major undergraduates, the findings suggest that career preparation in the context of AI warrants attention to both perceived replacement risks and uncertainty about personal career prospects. Career exploration could focus on specific fields, such as translation, education, and language services. Information about job tasks, competency requirements, and actual applications of AI could serve as reference material when considering career options. Students could review the preparation required for different career paths in light of their interests and current abilities. Occupational information gathering, setting short-term goals, and considering alternative career paths could also form part of this exploration.

For university career advisors, counselors, and coordinators of English-major programs, the findings suggest that discussions about AI and employment could also address concerns about future career stability and development prospects. Career guidance could explore changes in job roles, career options, and individual preparation needs in relation to specific student concerns. In addition, the positive association between PAIRT and CI was weaker at higher levels of CDMSE. This finding identifies confidence in career decision-making as an individual difference worth considering in employment support. Universities could explore support needs related to self-appraisal, occupational information gathering, goal selection, and planning when developing counseling content. AI-skills training could also be considered as a component of career preparation, with activities addressing tool use, output evaluation, and application boundaries in tasks relevant to English-related occupations. These are theoretically informed directions for support. Their effects on CI and employment anxiety remain to be evaluated in intervention studies.

For employers in English-related industries and organizations involved in university-industry partnerships, recruitment presentations, internship guidance, and career discussions could provide more specific information about job roles. Relevant topics include tasks involving AI, competency requirements, and potential career development paths. For example, task demonstrations and accounts of professional experience could illustrate how language proficiency, technical skills, and intercultural communication competence are used in different roles. Such information could serve as a reference for students considering career options and for universities reviewing how course content and internship arrangements relate to industry needs.

## Limitations and future research

First, the cross-sectional design does not allow the temporal ordering of PAIRT, CI, and employment anxiety to be established. Therefore, rather than being proof of causal or temporal links, the indirect and conditional indirect effects described in this study should be viewed as statistical associations. Future longitudinal or multi-wave studies could further examine how these relationships develop over time. Second, all focal variables were assessed using student self-reports. Although the analyses did not indicate serious common method bias, shared source and response tendencies cannot be completely excluded. Future research could incorporate data from multiple sources or complementary behavioral and career-related indicators. Third, the study used convenience sampling among English-major undergraduates in China. Despite the fact that participants were drawn from a variety of universities, the sample might not accurately reflect students majoring in English in various geographical areas, types of institutions, or educational settings. Future research should look at whether the results apply to other nations and educational systems using larger probability-based samples. Finally, PAIRT and the career replaceability dimension of employment anxiety share some item content concerning the potential replacement of future jobs or work tasks. Excluding this dimension yielded substantively similar results. Future research could further distinguish AI-specific replacement appraisals from broader employment anxiety using measures with more differentiated content.

## Conclusion

This study draws on CAT to examine the relationships among PAIRT, CI, CDMSE, and employment anxiety among English-major undergraduates. PAIRT was significantly and positively associated with employment anxiety and CI. A significant statistical indirect effect of PAIRT on employment anxiety through CI was observed. The positive association between PAIRT and CI was weaker at higher levels of CDMSE, and the corresponding conditional indirect association also varied significantly across levels of CDMSE. Overall, these findings extend the application of CAT to AI-related career contexts and provide insights for university career support for English-major undergraduates. The results should be understood as statistical connections rather than causal or temporal processes due to the cross-sectional design. Future longitudinal studies could further examine the temporal ordering of these relationships.

## Data Availability

The raw data supporting the conclusions of this article will be made available by the authors, without undue reservation.

## Footnotes

1
www.Sojump.com
