# Supplementary material for: The relationship between perceived AI replacement threat and employment anxiety among English-major undergraduates: the mediating role of career insecurity and the moderating role of career decision-making self-efficacy
Source: Front Psychol. 2026 Oct 27;17:1867329. doi: 10.3389/fpsyg.2026.1867329 (PMC13747789; doi:10.3389/fpsyg.2026.1867329)
Supplement: Supplementary file 1 [file Supplementary_file_1.docx]

# Appendix

Appendix Table S 1 Competing Measurement Models

| Model | *χ²* | *df* | *χ²/df* | CFI | TLI | RMSEA | AIC |
| --- | --- | --- | --- | --- | --- | --- | --- |
| Employment Anxiety | | | | | | | |
| One-factor model | 713.6 | 119 | 6.000 | 0.842 | 0.817 | 0.083 | 781.6 |
| Correlated four-factor model | 273.8 | 113 | 2.420 | 0.951 | 0.940 | 0.045 | 353.8 |
| Second-order model | 280.9 | 115 | 2.440 | 0.949 | 0.938 | 0.045 | 356.9 |
| Career Insecurity | | | | | | |  |
| One-factor model | 45.8 | 20 | 2.292 | 0.975 | 0.961 | 0.043 | 77.8 |
| Correlated two-factor model | 34.9 | 19 | 1.838 | 0.983 | 0.971 | 0.039 | 68.9 |

Appendix Table S 2 Comparison of Alternative Measurement Models

| Model | Specification | *χ²* | *df* | *χ²/df* | CFI | TLI | IFI | RMSEA |
| --- | --- | --- | --- | --- | --- | --- | --- | --- |
| Model 1 | Four-factor model | 3527.482 | 1341 | 2.630 | 0.910 | 0.904 | 0.911 | 0.048 |
| Model 2 | Perceived AI Replacement Threat and Employment Anxiety combined | 5022.571 | 1370 | 3.666 | 0.85 | 0.843 | 0.85 | 0.061 |
| Model 3 | Perceived AI Replacement Threat and Career Insecurity combined | 5124.632 | 1370 | 3.741 | 0.846 | 0.839 | 0.846 | 0.062 |
| Model 4 | Employment Anxiety and Career Insecurity combined | 5129.807 | 1370 | 3.744 | 0.846 | 0.839 | 0.846 | 0.062 |
| Model 5 | Perceived AI Replacement Threat, Employment Anxiety, and Career Insecurity combined | 5311.962 | 1372 | 3.872 | 0.838 | 0.831 | 0.839 | 0.064 |
| Model 6 | One-factor model | 9109.666 | 1373 | 6.635 | 0.682 | 0.669 | 0.683 | 0.089 |

Appendix Table S 3 Robustness Check With Additional Demographic Covariates

| Focal Effect | Primary Model | Fully Adjusted Model |
| --- | --- | --- |
| Perceived AI Replacement Threat → Career Insecurity | 0.743[0.597, 0.924] | 0.779[0.626, 0.967] |
| Career Insecurity → Employment Anxiety | 0.512[0.408, 0.637] | 0.445[0.353, 0.555] |
| Perceived AI Replacement Threat → Employment Anxiety | 0.406[0.275, 0.566] | 0.374[0.254, 0.523] |
| Indirect effect: Perceived AI Replacement Threat → Career Insecurity → Employment Anxiety | 0.380[0.285, 0.514] | 0.347[0.259, 0.467] |
| Perceived AI Replacement Threat × Career Decision-Making Self-Efficacy → Career Insecurity | -0.051[-0.092, -0.009] | -0.051 [-0.093, -0.010] |
| Index of moderated mediation | -0.027[-0.055, -0.002] | -0.027 [-0.055, -0.003] |

Appendix Table S 4 Comparison of Structural Path Estimates Between the Primary and Sensitivity Models

| Path | Primary model: 17-item Employment Anxiety | | | | Sensitivity model: 13-item Employment Anxiety | | | |
| --- | --- | --- | --- | --- | --- | --- | --- | --- |
|  | *Estimate* | SE | *p* | 95% CI | *Estimate* | SE | *p* | 95% CI |
| Perceived AI Replacement Threat → Career Insecurity | 0.743 | 0.076 | <0.001 | [0.597, 0.924] | 0.773 | 0.080 | <0.001 | [0.622, 0.963] |
| Career Insecurity →Employment Anxiety | 0.512 | 0.048 | <0.001 | [0.408, 0.637] | 0.455 | 0.044 | <0.001 | [0.357, 0.567] |
| Perceived AI Replacement Threat → Employment Anxiety | 0.406 | 0.058 | <0.001 | [0.275, 0.566] | 0.375 | 0.056 | <0.001 | [0.246, 0.543] |
| Gender → Career Insecurity | 0.092 | 0.066 | 0.160 | [-0.037, 0.221] | 0.100 | 0.071 | 0.160 | [-0.040, 0.241] |
| Gender →Employment Anxiety | 0.171 | 0.045 | <0.001 | [0.080, 0.276] | 0.193 | 0.046 | <0.001 | [0.101, 0.302] |
| Grade → Career Insecurity | 0.001 | 0.023 | 0.951 | [-0.043, 0.046] | 0.001 | 0.024 | 0.975 | [-0.047, 0.049] |
| Grade →Employment Anxiety | -0.010 | 0.015 | 0.517 | [-0.040, 0.017] | -0.004 | 0.015 | 0.785 | [-0.035, 0.024] |

Appendix Table S 5 Comparison of Direct, Indirect, and Total Effects Between the Primary and Sensitivity Models

| Effect | 17-item Employment Anxiety | 95% Bootstrap CI | 13-item Employment Anxiety | 95% Bootstrap CI |
| --- | --- | --- | --- | --- |
| Indirect Effect | 0.380 | [0.285, 0.514] | 0.351 | [0.260, 0.477] |
| Direct Effect | 0.406 | [0.275, 0.566] | 0.375 | [0.246, 0.543] |
| Total Effect | 0.787 | [0.627, 0.979] | 0.727 | [0.578, 0.914] |
| Indirect Effect Proportion | 48.29% | - | 48.28% | - |

Appendix Table S 6 First-Stage Moderation Analysis in the Sensitivity Model

| Predictor | *Estimate* | SE | *t* | *p* | LLCI | ULCI |
| --- | --- | --- | --- | --- | --- | --- |
| Perceived AI Replacement Threat | 0.435 | 0.033 | 13.077 | < 0.001 | 0.370 | 0.501 |
| Career Decision-Making Self-Efficacy | -0.127 | 0.033 | -3.780 | < 0.001 | -0.192 | -0.061 |
| Perceived AI Replacement Threat ×Career Decision-Making Self-Efficacy | -0.051 | 0.021 | -2.405 | 0.016 | -0.092 | -0.009 |
| Gender | 0.158 | 0.089 | 1.779 | 0.076 | -0.016 | 0.332 |
| Grade level | 0.001 | 0.031 | 0.017 | 0.986 | -0.059 | 0.060 |

Appendix Table S 7 Simple Slopes, Conditional Indirect Effects, and Index of Moderated Mediation in the Sensitivity Model

| Career Decision-Making Self-Efficacy Level | Perceived AI Replacement Threat → Career Insecurity | SE | 95% CI | *p* |
| --- | --- | --- | --- | --- |
| Simple Slopes of Perceived AI Replacement Threat Predicting Career Insecurity | | | | |
| -1 SD | 0.486 | 0.040 | [0.407, 0.565] | < 0.001 |
| Mean | 0.435 | 0.033 | [0.370, 0.501] | < 0.001 |
| +1 SD | 0.385 | 0.039 | [0.309, 0.461] | < 0.001 |
| Conditional Indirect Effects | | | | |
| Career Decision-Making Self-Efficacy level | Indirect effect | BootSE | BootLLCI | BootULCI |
| -1 SD | 0.248 | 0.029 | 0.193 | 0.304 |
| Mean | 0.223 | 0.026 | 0.173 | 0.273 |
| +1 SD | 0.197 | 0.029 | 0.137 | 0.252 |
| Index of Moderated Mediation | | | | |
| Moderator | Index | BootSE | BootLLCI | BootULCI |
| Career Decision-Making Self-Efficacy | -0.026 | 0.013 | -0.054 | -0.002 |
